# A Comparison of PlayerLoad^TM^ and Heart Rate during Backwards and Forwards Locomotion during Intermittent Exercise in Rugby League Players

**DOI:** 10.3390/sports9020021

**Published:** 2021-01-25

**Authors:** Matthew R. Barnes, Joshua H. Guy, Nathan Elsworthy, Aaron T. Scanlan

**Affiliations:** 1 School of Health, Medical and Applied Sciences, Central Queensland University, Rockhampton, QLD 4740, Australia; matthew.r.barnes@cqumail.com (M.R.B.); j.guy@cqu.edu.au (J.H.G.); 2 Human Exercise and Training Laboratory, Central Queensland University, Rockhampton, QLD 4702, Australia

**Keywords:** playerload, accelerometer, workload, heart rate, RPE, rugby

## Abstract

Limited research has examined the demands of backward locomotion at various speeds using common load monitoring metrics in team sport athletes. Consequently, this study compared the external and internal loads between backward and forward locomotion during intermittent exercise in team sport athletes. Semi-professional, male rugby league players (*n* = 29) completed the same exercise protocol on two occasions in backward and forward directions. On each occasion, participants performed separate 20 m trials at self-selected walking, jogging, running, and sprinting speeds and then completed a 15 min modified Loughborough intermittent shuttle test (mLIST). Common external and internal load metrics were gathered across testing. Faster speeds (*p* < 0.001) were attained at all speeds during forward locomotion in the 20 m trials. Non-significant differences in accumulated PlayerLoad^TM^ were found between directions across the mLIST; however, higher relative (per min) PlayerLoad^TM^ (*p* < 0.001) was apparent during backward locomotion when walking and during forward locomotion when sprinting during the mLIST. RPE and mean heart rate were higher (*p* < 0.001) during backward locomotion across the mLIST. These data highlight the unique loading patterns experienced during backward locomotion and suggest practitioners should consider the discernment in loading imposed between backward and forward locomotion when measuring athlete demands using common metrics.

## 1. Introduction

Many team sports require effective multi-directional, unorthodox movements during training and match-play [1,2,3,4]. While forward and lateral movements contribute to most sport locomotion, there are many circumstances where backward movement efficiency could be decisive in determining performance outcomes [3,5]. Backward locomotion is often performed in situations where an athlete is required to change direction while maintaining visual focus on the opposition player and/or ball ahead. For example, players in rugby codes often move in a backward direction when on defense while facing attacking players. This scenario is evident following a tackle with the defensive player performing backward locomotion for 3.6–5.4 m after each tackle, and this is often performed with high effort [3]. Importantly, it is suggested that elite soccer players performing significantly more backward running compared to lower-level players during matches [6]; however, there is no data specific to rugby league to support this.

Understandably, forward locomotion has received significant attention from high-performance staff and coaches in team sports when implementing training plans to develop power, strength, and speed attributes for optimal athlete performance. Despite the attention surrounding forward locomotion to optimize athletic performance, few studies have explored backward locomotion [7,8,9]. This lack of research is surprising given the contribution of backward locomotion to the overall match demands in various team sports. Furthermore, backward locomotion is important in the context of team sport performance and is a useful training strategy to prepare athletes for the multi-directional locomotive demands encountered during match-play [10,11,12].

A recent review by Uthoff et al. [7] summarized the acute physiological and biomechanical demands of backward running, concluding the cardiorespiratory responses and energetic requirements of backward locomotion are significantly higher than forward locomotion [7]. Specifically, heart rate and oxygen consumption have been shown to be ~15% higher during backward running than forward running at matched speeds (1.75–3.50 m·s^−1^) [13]. These increased energetic demands during backward locomotion are likely due to different muscle recruitment and activation patterns, as well as other biomechanical differences such as increased stride frequency and decreased knee joint loading compared to forward locomotion. Interestingly, exposure of athletes to backward locomotion is beneficial in various ways, such as reducing injury risk and aiding the rehabilitation process [12,14,15,16]. Specifically, Uthoff et al. [7] recently summarized a selection of programs incorporating backward running, and many have shown benefit to reducing lower limb injury prevalence. Neglect of backward running, therefore, may increase injury prevalence in many team sport athletes. However, the precise whole-body external load imposed during backward locomotion remains to be quantified using microsensor technology, which is a common monitoring approach adopted in many team sports.

Microsensors are widely used to provide an objective measure of external loading during training and match-play in team sports through the provision of various metrics. External loads refer to the amount of work an athlete has performed during a bout of exercise, measured independently of their internal characteristics (i.e., distance covered, power output, acceleration) [17]. Conversely, the internal load is a measure of the physiological/psychological stress imposed on the athlete (i.e., heart rate). PlayerLoad^TM^ is a popular metric obtained from microsensors and is calculated as the summation of changes in accelerations across the three-movement planes (transverse, coronal, and sagittal planes). An in-built tri-axial accelerometer and a unique algorithm to promulgate a measure in arbitrary units (AU) are used to quantify multi-directional external loading as PlayerLoad^TM^ [1,18,19]. The unique biomechanical characteristics of backward locomotion may therefore result in different external loads imposed on athletes during training and matches than forward locomotion. Furthermore, external loading profiles may vary according to movement speed, as it has been established that speeds achieved during forward locomotion are approximately 30% greater than that achieved during backward locomotion [7]. Understanding the differences in external loading between forward and backward locomotion is important for the continued implementation of backward-oriented drills as training stimuli in team sport environments [7]. It is also important to examine and compare the internal loads encountered during backward and forward locomotion at different speeds to understand if similar dose–response relationships exist between external and internal metrics when moving in different directions. As such, this study will quantify and compare the external and internal loads during backward and forward locomotion during intermittent exercise in team sport athletes.

## 2. Materials and Methods

### 2.1. Participants

Semi-professional, male rugby league players (*n* = 29; age: 25.2 ± 3.5 year; body mass: 76.5 ± 8.4 kg; height: 179.7 ± 5.7 cm) completed two testing sessions during the pre-season phase of the annual training plan. Participants were recruited from the same rugby league club competing in a state-based, sub-elite Australian rugby league competition. All participants provided written informed written consent prior to involvement in the study and were free from injury or medical conditions that contraindicated participation. All study procedures were approved by the Central Queensland University Human Research Ethics Committee (approval no. 0000021338). A power analysis was performed a priori whereby a sample of 27 subjects was estimated to detect a moderate effect (dz = 0.5) with a power of 0.8 using a paired *t*-test between means with alpha at 0.05.

### 2.2. Procedures

Using a randomized, crossover design, participants completed two testing sessions separated by 48 h. Participants avoided strenuous exercise for 3 days leading into the first testing session and the 2 days between testing sessions. Each session consisted of a standardized warm-up, self-selected baseline speed testing at four different speeds, and a 15 min bout of a modified Loughborough intermittent shuttle test (mLIST). Both sessions were identical; however, baseline speed testing and the mLIST were performed in a backward direction in one session and a forward direction in the other session. All tests were completed on a flat grass surface on the same rugby league field, and all players were familiarized with the protocols prior to the study.

Each participant was fitted with a microsensor containing an accelerometer (100 Hz, OptimEye S5, Catapult Innovations; Melbourne, Australia) prior to testing. Microsensors were placed in tight-fitting neoprene vests according to manufacturer guidelines and positioned between the scapulae of each participant to align with typical microsensor positioning during training and match settings. A chest-worn heart rate monitor (T31, Polar Electro; Kempele, Finland) was also fitted to each participant throughout testing.

A 5 min standardized warm-up was administered prior to activity in both testing sessions, consisting of jogging, 20 m runs at progressively higher speeds, and dynamic stretching. Immediately following the warm-up, baseline self-selected speed testing was conducted with performance times measured using an electronic timing light system (Fusion Sport, Coopers Plains, Australia). This system is a single-beam photoelectric timing light system and has a typical error of <0.03 s [20]. Gates were set with the beam positioned at the height of 1 m from the ground on a tripod [21]. Each participant performed two trials at each of the four speeds (walking, jogging, running, and sprinting) to determine the average speed attained at each speed in each movement direction. Movement speeds were instructed to be performed relative to the perceived maximal effort (% _ME_) and were self-selected by participants, including walking at normal walking speed, jogging at 40–55% _ME_, running at 65–75% _ME_, and sprinting at >90% _ME_ [9]. Speeds were randomized for each participant, with 2 min of passive standing recovery provided between each trial. Speed testing data were downloaded from the SmartSpeed^TM^ cloud-based data storage system, and stored in Microsoft Excel (v15.0, Microsoft Corporation, Redmond, WA, USA). The average speed (m·s^−1^) for each speed category across 20 m was calculated from the total time achieved.

**Figure 1:**
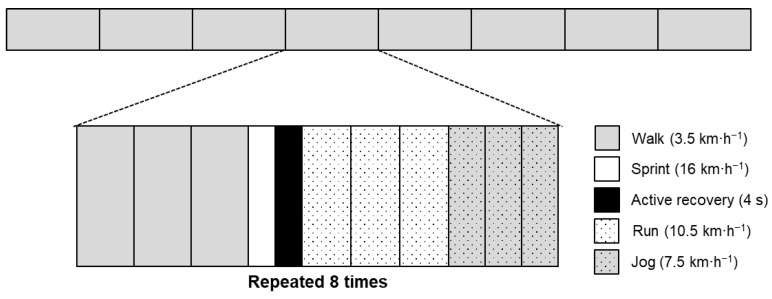
Schematic of the modified Loughborough intermittent shuttle test.

Upon completion of the 20 m trials, the mLIST was performed. The mLIST was ~15 min in duration, with eight identical blocks of activity performed. Each activity block consisted of 3 × 20 m walking bouts (3.5 km·h^−1^), 1 × 20 m sprinting bout (16 km·h^−1^; followed by 4 s of passive standing recovery), 3 × 20 m running bouts (10.5 km·h^−1^), and 3 × 20 m jogging bouts (7.5 km·h^−1^) with the pace of each bout dictated by audio cues. The assigned speeds were identical for both directions, and to enable the test to be completed successfully, selected speeds were appropriate for the backward direction [9]. Using the Team Beep Test iPhone application, the audio file was created using the assigned speeds for each type of displacement. A schematic of the mLIST is presented in Figure 1. Within 30 min of completing the mLIST, rating of perceived exertion (RPE) was collected from each participant using the 6–20 scale [22]. Accelerometer and heart rate data were downloaded from the microsensors using proprietary software (Catapult Sprint, v5.1.7; Catapult Innovations; Melbourne, Australia). Data were then manually separated according to the activity (walking, jogging, running, and sprinting) completed during the mLIST. The external load was determined using accumulated PlayerLoad^TM^, which has been described as the sum of a modified vector magnitude calculated as the square root of the sum of the squared instantaneous rate of change in acceleration across three planes multiplied by a scaling factor of 0.01 [23]. PlayerLoad^TM^ relative to time (AU·min^−1^) was also determined for each phase of the mLIST given the differences in duration spent at each movement type during specific phases of the mLIST. The accelerometer devices used in this study have been shown to be reliable in a team sport with a coefficient of variation of less than 2% [23]. Internal load variables included RPE and mean heart rate (HR_mean_) for the entire mLIST. The validity and reliability of accelerometry-, heart rate-, and RPE-based load variables have been rated as moderate-high in a consensus statement [24].

### 2.3. Statistical Analysis

All data were assessed for normality using the Shapiro–Wilk test, and visually examined using Q–Q plots and were found to be normally distributed (*p* > 0.05). All data were calculated as mean ± standard deviation. Comparisons in each speed (walking, jogging, running, and sprinting) obtained during the 20 m trials, as well as HR_mean_, RPE, and accumulated PlayerLoad^TM^ across the entire mLIST, were made between backward and forward locomotion using paired sample *t*-tests. Statistical significance was accepted when *p* ≤ 0.05. To examine differences in relative PlayerLoad^TM^ for each speed during the mLIST (walking, jogging, running, and sprinting), additional paired sample *t*-tests were performed between backward and forward locomotion with Bonferroni corrections made to control for Type I errors (*p* < 0.0125). Cohen’s effect sizes (ES) with 95% confidence intervals (CI) were calculated for pairwise comparisons in all variables, with effect magnitudes interpreted as: *trivial* (<0.20); *small* (0.20–0.59); *moderate* (0.60–1.19); *large* (1.20–1.99); and *very large* (≥2.0) [25]. If the 95% CI of an effect crossed the boundaries of ±0.2, the effect was interpreted as *unclear* [25]. Statistical analyses were performed in SPSS (v26) and Microsoft Excel (v15.0).

## 3. Results

**Figure 2:**
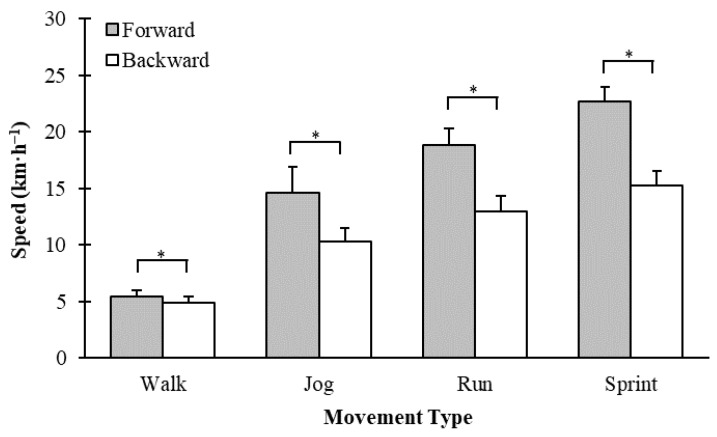
Average linear speeds (mean ± standard deviation) attained during self-selected walking, jogging, running, and sprinting movements in backward and forward directions. * indicates a significant difference between forward and backward trials for each movement type.

The mean ± standard deviation speeds attained during the initial, linear self-selected speed testing are shown in Figure 2. Pairwise comparisons in self-selected speeds revealed faster speeds were attained during forward locomotion compared to backward locomotion in all movement types (*p* < 0.001) (Figure 2). At each self-selected speed, the average speed for backward locomotion was 8.7 ± 11.0%, 27.7 ± 14.9%, 30.9 ± 8.5%, and 32.8 ± 4.9% slower than forward locomotion for walking, jogging, running, and sprinting, respectively.

The mean ± standard deviation external and internal loads encountered in each locomotion direction across the mLIST protocol are shown in Table 1. While accumulated PlayerLoad^TM^ was not significantly different between locomotion directions (*p* = 0.63; d = 0.11 [95% CI = −0.27 to 0.49], *unclear*), significant differences emerged when examining relative PlayerLoad^TM^ at different speeds during the mLIST. Specifically, relative PlayerLoad^TM^ was significantly higher during backward walking compared to forward walking (*p* < 0.001; d = −1.04 [95% CI = −1.42 to −0.66], *moderate*) and during forward sprinting compared to backward sprinting (*p* < 0.001; d = 0.76 [95% CI: 0.38 to 1.14], *moderate*) (Table 1). Regarding internal load variables, significant differences in RPE (*p* < 0.001; d = −2.39 [95% CI = −2.77 to −2.01] *very large*) and HR_mean_ (*p* = 0.01; d = −0.52 [95% CI = −0.90 to −0.14], *small*) were identified with higher responses evident during backward compared to forward locomotion across the mLIST.

## 4. Discussion

This study quantified and compared the external and internal loads encountered between backward and forward locomotion during intermittent exercise in rugby league athletes. The main findings were: (1) significantly faster self-selected speeds ranging from walking to sprinting paces were evident during forward compared to backward locomotion; (2) no significant difference in accumulated PlayerLoad^TM^ was apparent between backward and forward locomotion across the mLIST; (3) significantly higher relative PlayerLoad^TM^ was evident during backward walking compared to forward walking, and during forward sprinting compared to backward sprinting during the mLIST; and (4) internal loads (RPE and HR_mean_) were significantly greater during backward compared to forward locomotion across the mLIST.

It is well established that maximal speeds achieved during forward sprinting are higher compared to backward sprinting [7]. In this study, higher self-selected speeds were achieved for all movement types in forward compared to backward locomotion, with speed reached during backward sprinting being 67 ± 5% of that achieved during forward sprinting. This finding adds to the limited body of research on this topic and provides foundation evidence in a large group of competitive team sport athletes. Indeed, previous research data demonstrating backward running speeds were ~70% of forward running speeds in high-school athletes [9] and active participants [26] align with the current findings in semi-professional, rugby league players. Forward sprinting takes advantage of the stretch-shortening cycle and the propulsive forces of the plantar flexors, hamstrings, and gluteal muscles, enabling high force production and therefore speed. However, during backward running, the role of these muscles switches to attenuate braking forces, while the anterior muscles (i.e., quadriceps and tibialis anterior) are responsible for the propulsive forces [6,27]. These differences in muscle recruitment patterns are likely responsible for the observed differences in locomotion speed between directions when performing at near-maximal speeds. Nevertheless, backward locomotion remained slower than forward locomotion at the same self-selected speed during submaximal movements (walking, jogging, and running). In this regard, previous research suggests the motor programs responsible for coordinating backward and forward locomotion may be the same; however, the slower speeds performed during backward locomotion may be due to greater movement variability in this direction [28]. When walking or running backward, participants may compensate for movement speed to ensure a successful and coordinated movement, particularly given the lack of visual information available to assist with speed control and foot placement when facing backward. In addition to supporting the limited available evidence already provided in active participants showing superior speeds are attained during forward locomotion compared to backward locomotion across various speeds [9,26], this study provides the first insight into how variations in movement speeds between backward and forward directions translate to a common metric used to monitor external load in team sport athletes, PlayerLoad^TM^.

No significant difference between locomotion directions was evident for accumulated PlayerLoad^TM^ across the entire mLIST. However, differences became apparent between directions when specific movement phases predicated on speed were examined in isolation during the mLIST. Specifically, relative PlayerLoad^TM^ was higher in forward sprinting compared to backward sprinting during the mLIST, potentially due to forward sprinting promoting higher vertical forces than backward sprinting [26]. The location of the center of pressure during the gait cycle may also underpin our findings being positioned closer to the toes during backward sprinting and attenuating vertical forces through the foot and ankle complex [7]. These combined factors may translate into a greater external loading being detected via accelerometry when running in a forward direction. However, the heightened relative PlayerLoad^TM^ during forward locomotion was only evident at sprinting speed (16 km·h^−1^), and in turn, these findings were reversed during walking, with relative PlayerLoad^TM^ higher during backward walking compared to forward walking. This trend may relate to the mechanical work involved during backward walking, where Minetti and Ardigò [29] showed 48% higher mechanical work was exerted during backward walking compared to forward walking at 4 km·h^−1^, which was similar to the walking speeds assessed in the present study (5 km·h^−1^). Furthermore, walking backward is more difficult and demanding to accomplish than walking forward due to the postural instability, and the lack of visual feedback encountered [30]. To compensate for these factors, decreased stride length, cadence, and speed occur during backward walking compared to forward walking, potentially augmenting the relative PlayerLoad^TM^ during backward locomotion across walking bouts throughout the mLIST. Cadence or stride length, however, were not recorded in the present study and future studies are encouraged to measure these biomechanical, mechanistic variables when comparing PlayerLoad^TM^ between backward and forward locomotion.

The energetic demands of backward locomotion have been consistently shown to be higher compared to forward locomotion at similar speeds [6,27,31,32]. For instance, backward locomotion elicits a 28% increase in oxygen consumption and a 15% increase in heart rate compared to forward locomotion at matched speeds (2.24 m·s^−1^) [33]. The present data concur with these findings demonstrating similar differences in heart rate between movement directions in a large group of team sport athletes. In this way, increased reliance on knee extensor motor unit recruitment is required to generated propulsion during backward locomotion [31,34]. The greater muscle activation and step frequency during backward locomotion likely increase the oxidative energy requirements of the task with concomitant increases in heart rate compared to forward locomotion. Furthermore, internal perceptual loading was also significantly higher during backward compared to forward locomotion, suggesting participants were required to perform at a greater level of exertion to maintain pace with the audio cues. While the collective evidence indicates internal loading is higher during backward locomotion than forward locomotion [see 6 for a summary], the relationship between external and internal loads according to movement direction presents unique but important practical implications.

Typically, greater external loads will increase the resultant internal loads due to the heightened metabolic cost and force production of soft tissue [35]. However, our data show additional factors (movement direction) can impact this relationship and potentially mask specific loading responses. The higher internal loading combined with, the lower external loading at high speeds during backward running in the present study may be interpreted as abnormal responses to the imposed physical stimuli (i.e., elevated internal load relative to a given external load) when in fact, it is representative of direction-specific task demands. Therefore, when examining athlete responses to training or match stimuli where higher than normal backward locomotion may have been performed, the external and internal loads may not follow the expected patterned outcomes. Furthermore, when interpreting external load data using a common metric in PlayerLoad^TM^, loads may be augmented during activities involving backward locomotion at lower speeds and reduced during activities involving backward locomotion at higher speeds. Consequently, these variable loading patterns should be considered when making informed decisions regarding the loading undertaken by rugby league players where backward locomotion is regularly performed.

The backward movement of rugby league players is most evident during defensive scenarios whereby players are required to retreat 10 m following a tackle. The laws of the game dictate that players must be at least 10 m from the play the ball. It is important at this time that players maintain a vision of the attacking team in these instances in order to prepare for the next act of play. Therefore, at these times, players often make a concerted effort to retreat to the 10 m line while also ensure they have a visual focus on the attacking players. As this study has shown, there are differences in the PlayerLoad^TM^ and heart rate responses between forward and backward running at match speeds. These findings are important for the preparation and monitoring procedures of rugby league practitioners. Backward running has benefits for rugby league players in the preparation of games and prevention of injury, while these findings provide a reference for the differences in the loading of backward movement performed in rugby league. While this study provides novel data comparing the loads encountered during backward and forward locomotion in team sport athletes and using common load monitoring metrics, there were some limitations faced. First, the external load was measured via torso-mounted microsensors. As such, the specific loading imposed at different sites, such as the waist or lower limbs, may yield different results to those observed in the present study. For instance, greater muscle activation in the quadriceps, hamstrings, gastrocnemius, and tibialis anterior muscles during backward running may exacerbate loading responses when measured at the lower limbs. Future studies should examine external loading measured at different body locations using modern microtechnology to comprehensively understand the demands of backward locomotion. Second, internal loads at different velocities were not able to be isolated during the mLIST. In turn, future research implementing steady-state exercise protocols at different speeds may provide more detailed analyses of the relationship between external and internal loads at different velocities according to the movement direction. Third, given the phases of the mLIST were performed in a repetitive, cyclic manner, the demands of previous activity may have impacted the loading imposed on athletes during subsequent phases. For example, following each sprint, three 20 m run bouts were performed, and as such, the overall “run” external load could have been influenced by residual fatigue induced from the sprinting activity.

## 5. Conclusions

The overall external loading imposed on rugby league athletes during backward locomotion during intermittent exercise was not significantly different from those imposed during forward locomotion. However, external loading varied between movement directions during activities at specific speeds, with forward sprinting producing greater external loads compared to backward sprinting and backward walking producing greater external loads than forward walking. The internal loading experienced during intermittent backward locomotion was significantly higher than forward locomotion. Consequently, backward locomotion appears to elicit higher internal responses in rugby league athletes at various movement speeds but produces lower external loading at higher speeds and a higher external loading at lower speeds compared to forward locomotion. Rugby league practitioners should be aware of these unique external and internal loading patterns during backward and forward movement directions when interpreting and managing athlete loads across the season.

## Figures and Tables

**Table 1:** Internal and external loads (mean ± standard deviation) imposed during the modified Loughborough intermittent shuttle test in forward and backward movement directions.

Variable	Movement Direction			Statistical Outcomes	
	Forward	Backward	Mean Difference	Effect Size (95% CI)	*p*
Internal load					
RPE (AU)	12.4 ± 2.2	16.1 ± 2.1	3.7 ± 1.6	−2.39 (−2.77 to −2.01)	<0.001
HR_mean_ (beats·min^−1^)	155.5 ± 13.1	162.4 ± 14.2	6.9 ± 13.5	−0.52 (−0.90 to −0.14)	0.01
External load					
Accumulated PlayerLoad^TM^ (AU)	151.9 ± 14.0	149.9 ± 18.0	−1.9 ± 17.0	0.11 (−0.27 to 0.49)	0.63
Relative PlayerLoad^TM^ (AU·min^−1^)					
Walk	2.8 ± 0.5	3.4 ± 0.5	0.7 ± 0.6	−1.04 (−1.42 to −0.66)	<0.001
Jog	12.5 ± 1.1	12.3 ± 1.6	−0.2 ± 1.4	0.17 (−0.21 to 0.55)	0.37
Run	16.9 ± 1.8	16.0 ± 2.1	−0.9 ± 2.2	0.41 (0.03 to 0.79)	0.04
Sprint	30.6 ± 3.3	26.9 ± 4.8	−3.8 ± 5.0	0.76 (0.38 to 1.14)	<0.001

Note: CI = confidence intervals; RPE = rating of perceived exertion; HR_mean_ = average heart rate; AU = arbitrary units.

## Data Availability

The data presented in this study are available on request from the corresponding author.
